# Quality of life of parents of very preterm infants 4 months after birth: a mixed methods study

**DOI:** 10.1186/s12955-018-1011-y

**Published:** 2018-09-10

**Authors:** Mariana Amorim, Elisabete Alves, Michelle Kelly-Irving, Ana Isabel Ribeiro, Susana Silva

**Affiliations:** 1 EPIUnit - Instituto de Saúde Pública, Universidade do Porto, Rua das Taipas, n° 135, 4050-600 Porto, Portugal; 2 Departamento de Ciências da Saúde Pública e Forenses e Educação Médica, Faculdade de Medicina, Universidade do Porto, Al. Prof. Hernâni Monteiro, 4200 - 319 Porto, Portugal; 3 Global Public Health Doctoral Programme, Porto, Portugal; 4 INSERM UMR1027, F-31000 Toulouse, France; 5 Université Toulouse III Paul Sabatier, UMR1027, F-31000 Toulouse, France

**Keywords:** Quality of life, Parents, Very preterm birth, Family-integrated care, Mixed methods

## Abstract

**Background:**

Knowledge about parental quality of life (QoL) is paramount to family-centred and integrated healthcare on prematurity, but evidence is limited. We aimed to explore mothers’ and fathers’ perspectives about their QoL 4 months after a very preterm childbirth.

**Methods:**

This is a cross-sectional mixed methods study using a convergent design. Parents of very preterm infants were systematically recruited at all level III neonatal intensive care units in the Northern health region of Portugal for one year. Four months after childbirth, 61 mothers and 56 fathers filled-in the World Health Organization Quality of Life – BREF Inventory, and 26 couples were interviewed. Linear regression models were computed to assess the association between participants’ characteristics and the QoL. Qualitative data were thematically analysed.

**Results:**

A quantitative analysis revealed that the perception of QoL was not significantly different by gender. QoL scores increased slightly from the environment (Mean (SD): 72.1 (14.2)) to the psychological domains (Mean (SD): 78.7 (14.4)). All scores were influenced by psychological characteristics. Socioeconomic position influenced both parents’ perceptions concerning the environment domain, and maternal physical and psychological QoL. Infant-related factors were associated with overall QoL among women and with the physical, psychological, social and environment domains among men. Qualitative findings indicated accommodation mechanisms that intertwine the focus on constraining factors (surveillance, sleep disturbances, non-supportive healthcare policies, hygienization) with facilitating factors (social support, accessibility/quality of healthcare, opportunities for developing parental skills). These processes were anchored in child-centredness and a framework that construct hierarchies of hope and expectations about infant’s health and development.

**Conclusions:**

To capture parental QoL using mixed methods raises awareness for developing intersectoral family-centred policies, integrated health services and focused-interventions to decrease the disempowering effects of surveillance and hygienization.

## Background

Preterm birth is a major public health issue. Its complications constitute one of the leading causes of global deaths among children under 5 years of age [1], and preterm infants are at high risk of neonatal morbidity [2]. Globally, the average preterm birth rate in 2010 was estimated at 11.1%, corresponding to more than one in ten of all births [3], and about 1% were a very preterm birth, occurring before 32 gestational weeks [4]. Despite medical and technological advances, infants born very preterm remain at high risk of death and neurodevelopmental impairment, with studies revealing an average of crude in-hospital mortality rates of 14.2% in 10 European regions [5, 6]. A very preterm delivery and the ensuing child’s hospitalization in a Neonatal Intensive Care Unit (NICU) is considered a disruptive and stressful life event, affecting parental QoL via multiple pathways [7, 8], in a context with wide differences between the support to family-friendly and gender-equality policies in Western and Nordic European countries [9].

The literature consistently shows that the quality of life (QoL) of parents of preterm infants may be compromised by sleep disturbances, fatigue, stress and psychiatric symptoms [10–14], while being protected by a stable marital union, support and information provided by medical staff, partner, extended family and other parents of preterm children [14, 15]. This knowledge is essential to develop family-centred and integrated healthcare services and policies on prematurity [16, 17], an approach with benefits for parents, children and their families [18, 19] as well as for healthcare staff and health services [20].

However, studies exploring the intertwining of constraining and protective factors in the perception of QoL are scarce and focused on patients with chronic conditions [21]. Furthermore, there is still limited evidence about the impact of a preterm delivery on parental QoL, in a context where methodological heterogeneity is observed regarding the operationalization of QoL and the use of units of analysis (mothers, parents, families and caregivers) [22]. Finally, the influence of fathers’ characteristics and structural factors (e.g., parental leave policies) on parental QoL after a preterm delivery has not been sufficiently addressed in previous research [22].

Further studies are thus needed to explore both maternal and paternal QoL, in the analysis of the individual, familial and societal factors influencing QoL. Such in-depth parental perspectives are key, especially during the return-to-work period, which is a relevant moment in countries where few attempts are being made to support parental leave [23, 24]. A mixed methods approach would provide a more complete comprehension of the QoL questionnaires’ scores, contributing to accurately capture the singular experience of parenting a very preterm infant [25] and the complexity of QoL assessment [26]. By integrating quantitative and qualitative data, this study aims to explore mothers’ and fathers’ perspectives about their own QoL, 4 months after a very preterm delivery.

## Methods

This observational and cross-sectional mixed methods study used a convergent design aiming to merge quantitative and qualitative data into one overall interpretation, in which the quantitative results were validated or expanded with the qualitative data [27, 28]. This single-phase design (i.e. the quantitative and qualitative methods were implemented during the same timeframe and with equal weight) was chosen with the intention to best understand the QoL of parents of very preterm infants during the return-to-work period, ending up with well-substantiated conclusions about the factors that influence such phenomenon.

Between July 2013 and June 2014, all mothers and fathers of very preterm infants, admitted to all level III NICU located in the Northern Health Region of Portugal (*n* = 7), were consecutively and systematically invited to participate in the study by the healthcare team, 15 to 22 days after delivery. Parents who were present in the NICU during the hospitalisation period, who were able to speak and write in Portuguese, and those whose single or twin infants survived were considered eligible to participate in the study [29]. Among the 122 families invited, 96% agreed to participate in the evaluation at 4 months after delivery, the common return-to-work period in Portugal, in particular for mothers.

Clinical records were reviewed to retrieve data on pregnancy complications, multiple pregnancy, and infant’s gestational age and birth weight. Extremely low birth weight was defined as birth weight bellow 1000 g and extremely premature infants were those with gestational age under 28 weeks [4, 30].

### Quantitative study: Participants and data collection

Parents were contacted 4 months after delivery to confirm the availability to receive the questionnaires at home. Parents whose infants were still hospitalized (*n* = 1) or died (*n* = 3) were excluded from the study. Self-administered questionnaires to be completed individually, with prepaid return envelops, were sent by postal mail to 113 families. Among these, 67 mothers and 64 fathers completed and returned the questionnaires between November 2013 and November 2014 (Median months after childbirth (P25-P75): 4.3 (4.0–4.6)). After exclusion of the participants with > 20% of missing values on the QoL questionnaire, as recommended [31], 61 mothers and 56 fathers were included in the quantitative analysis.

Perceived QoL was assessed using the Portuguese version of the World Health Organization Quality of Life – BREF Inventory (WHOQOL-BREF) [32]. It is organized into a facet of *overall QoL* (general perception of QoL and health) and 4 domains: *physical* (pain and discomfort; energy and fatigue; sleep and rest; dependence on medication; mobility; activities of daily living; working capacity), *psychological* (positive and negative feelings; self-esteem; thinking, learning, memory and concentration; body image; spirituality, religion and personal beliefs), *social relationships* (personal relations; sexual activity; social support), and *environment* (financial resources; information and skills; recreation and leisure activities; home environment; accessibility and quality of health and social care; physical safety and security; physical environment; transport).

Data on sociodemographic characteristics were collected, as well as data regarding infants’ length of stay in NICU and the presence of health problems. Occupations were classified according to the Portuguese Classification of Occupations 2010 [33] and grouped in three categories: upper-white-collar, including executive civil servants, industrial directors and executives, professionals and scientists, middle management and technicians; lower-white-collar, including administrative and related workers, service and sales workers; and blue-collar, which includes farmers and skilled agricultural, fisheries workers, skilled workers, craftsmen and similar, machine operators and assembly workers, unskilled workers. Unemployed (*n* = 15) or retired participants (*n* = 1) were classified considering their previous main occupation.

Symptoms of anxiety, depression and parenting stress were assessed through Portuguese versions of The Beck Anxiety Inventory [34], the Beck Depression Inventory-II [35], and The Parenting Stress Index (PSI) [36], respectively.

Participants were georeferenced according to the home address, using the ArcGIS Online World Geocoding Service and Google Maps. Each participant was matched to the urbanity level [37] and the neighbourhood socioeconomic deprivation, assessed through The European Deprivation Index [38].

### Statistical analysis

Missing values of the WHOQOL-BREF inventory were replaced by means of the remaining domain items, when ≤ 2 items were missing from the domains *physical, psychological* and *environment* and 1 item in the *social relationships* domain [31]. Regarding the BDI and BAI scores, participants with > 2 items missing were discarded from the current analysis; the remaining missing values were replaced by the mean value for each item [35]. Missing values in the PSI were substituted using the subscale items if no more than 5 items from total scale, 3 items from each domain and 1 item from each subscale were missing [39].

The analysis was performed using Stata 11.0 (College Station, TX, 2009). The chi-square test and the t-test or the Mann-Whitney-test were used as appropriate. Linear regression models, stratified by gender, were computed to assess the association between participants’ characteristics and the QoL. Statistical significance was set at a value of *p* < .05.

### Qualitative study: Participants and data collection

Semi-structured qualitative interviews were conducted with a sub-sample of 26 couples between November 2013 and April 2014. Participants were purposively sampled to include parents of infants with extremely (< 1000 g) and non-extremely (≥ 1000 g) low birth weight. A heterogeneity sampling was used for maximum variation of views and experiences, until reaching thematic saturation. Therefore, recruitment continued until no new themes emerged from the interview data [40].

Interviews were conducted at parents’ home (*n* = 19), at the university department responsible for the study (*n* = 6) and in a private hospital room (*n* = 1). Interview duration ranged from 20 to 72 min (Mean: 39 min). All interviews were audio taped and transcribed verbatim. The interview guide covered the following areas: how parents deal with uncertainty and doubts and how they made their decisions concerning parental care, treatment options and uses of information sources; their views of the consent procedures; their understandings of medical facts, of technologies applied to perinatal care and of prognosis; their views of life and living with handicaps; information and communication needs of parents; and awareness of social and ethical issues in this area. Data related to parents’ perceptions of their QoL will be discussed by exploring the entire content of each interview.

### Content analysis

Thematic content analysis [41] was performed using the software NVivo 11 (QSR International, USA, 2015). A triangulation strategy was used to guarantee the rigour and quality of research - the first author identified, sentence by sentence, parents’ perceptions about the factors influencing (positively and negatively) their QoL after a very preterm delivery, and the last author collaborated on the development of the coding framework. Firstly, quotations with similar meanings were synthesized into categories, both deductively, in accordance with the facets of the WHOQOL-100 inventory [31], and inductively for the remaining data. Secondly, the categories were grouped into the following analytical themes: the domains of the WHOQOL-BREF inventory (Physical, Psychological, Social relationships and Environment) [31] and “Accommodation mechanisms”, corresponding to behavioural, cognitive, and emotional processes to accommodate a very preterm delivery [21]. The re-examination of qualitative data was performed when disagreements with quantitative results were found. The most illustrative verbatim quotes were selected by two authors and revised by an English native speaker.

## Results

The characteristics of the parents who completed the questionnaire and their association with QoL are presented in Tables 1 and 2, respectively. The results are explored integrating quantitative and qualitative data, according to QoL domains.

**Table 1:** Characterization of the participants who filled in the questionnaire, according to gender

	Total *n* = 117	Mothers *n* = 61	Fathers *n* = 56
Age < 35 years, n (%)	71 (62.8)	42 (68.9)	29 (55.8)
Educational level ≤ 12 years, n (%)	69 (60.5)	34 (55.7)	35 (66.0)
Married/living with a partner, n (%)	105 (92.1)	56 (91.8)	49 (92.5)
Occupation^a^, n (%)			
Upper white collar	46 (42.2)	22 (37.9)*	24 (47.1)*
Lower white collar	32 (29.4)	25 (43.1)*	7 (13.7)*
Blue Collar	31 (28.4)	11 (19.0)*	20 (39.2)*
Low/Medium-low subjective social class, n (%)	87 (77.7)	43 (71.7)	44 (84.6)
Neighbourhood socioeconomic deprivation, n (%)			
T1 (Least deprived)	50 (42.7)	27 (44.3)	23 (41.1)
T2	38 (32.5)	20 (32.8)	18 (32.1)
T3 (Most deprived)	29 (24.8)	14 (23.0)	15 (26.8)
Urbanity Level, n (%)			
Predominantly Rural/Moderately Urban	15 (12.8)	8 (13.1)	7 (12.5)
Predominantly Urban	102 (87.2)	53 (86.9)	49 (87.5)
Parenting stress			
Total stress scale^b^, Median (P25-P75)	216.5 (189.0–247.0)	220.0 (204.0–245.0)	209.0 (188.0–254.0)
Stressful life events scale^c^, Median (P25-P75)	10.0 (4.0–15.0)	11.0 (4.0–19.0)	10.0 (4.0–15.0)
Anxiety^d^, Median (P25-P75)	3.0 (1.0–7.0)	3.0 (1.0–7.7)	2.0 (1.0–5.0)
Depression^e^, Median (P25-P75)	4.0 (2.0–8.0)	6.0 (3.0–9.0)*	3.5 (1.0–6.0)*
Previous children, n (%)	29 (26.1)	16 (26.2)	13 (26.0)
Multiple pregnancy, n (%)	23 (19.7)	12 (19.7)	11 (19.6)
Pregnancy complications^f^, n (%)	51 (43.6)	27 (44.3)	24 (42.9)
Extremely low birth weight delivery^g^, n (%)	33 (28.2)	18 (29.5)	15 (26.8)
Extremely preterm delivery^h^, n (%)	24 (20.5)	13 (21.3)	11 (19.6)
NICU length of stay < 2 months, n (%)	71 (61.7)	37 (61.7)	34 (61.8)
Infants’ health problems^i^, n (%)	25 (21.4)	15 (24.6)	10 (17.9)
Quality of life (WHOQOL-BREF)^j^			
Overall, Mean (SD)	73.7 (12.4)	74.6 (12.5)	72.8 (12.4)
Physical domain, Mean (SD)	77.1 (12.6)	75.9 (12.2)	78.3 (13.1)
Psychological domain, Mean (SD)	78.7 (14.4)	77.2 (14.8)	80.4 (13.9)
Social relationships domain, Mean (SD)	75.1 (17.1)	75.8 (17.9)	74.3 (16.4)
Environment domain, Mean (SD)	72.1 (14.2)	72.9 (13.9)	71.3 (14.6)

^a^Students, housewives and armed forces occupations were excluded; ^b^The total stress score is the sum of the scores in two domains: child’s characteristics and parent’s characteristics, with higher scores indicating higher levels of parental stress (range for the total scale: 104 to 517); ^c^Stressful Life Events scale is composed by 24 different life events likely to cause stress (e.g.: unemployment, divorce, death of a relative), with higher values indicating more stress in life (range for the total scale: 0 to 114); ^d^Higher values indicate higher levels of anxiety symptoms (range for the total scale: 0 to 63); ^e^Higher values indicate higher levels of depressive symptoms (range for the total scale: 0 to 63); ^f^Infectious, placental, haemorrhagic and cardiovascular complications; ^g^ < 1000 g; ^h^ < 28 gestational weeks; ^i^Inguinal and umbilical hernias, metabolic disease, ovarian cysts, bronchial dysplasia, autoimmune disease, cardiac disease, congenital malformation; ^j^Higher values represent better QoL (Range: 0–100)

Notes: In each variable, the total may not add 117 parents, 61 mothers or 56 fathers due to missing values; The proportions may not add 100 due to rounding; SD, Standard Deviation; *p value < .05 for the comparison between mothers and fathers

**Table 2:** Crude association between characteristics of participants and quality of life, according to gender

	Quality of life (WHOQOL-BREF)									
	Mothers (*n* = 61) Crude b (95%CI)					Fathers (*n* = 56) Crude b (95%CI)				
	Overall QoL	Physical	Psychological	Social relationships	Environment	Overall QoL	Physical	Psychological	Social relationships	Environment
Age, years (< 35 vs. ≥35)	− 0.6 (− 7.6; 6.4)	− 1.7 (− 8.5; 5.1)	0.6 (− 7.7; 8.8)	−3.3 (− 13.2; 6.7)	0.1 (− 7.7; 7.8)	1.5 (− 5.6; 8.7)	1.8 (− 5.4; 9.1)	− 0.9 (− 8.3; 6.5)	1.9 (− 7.2; 10.9)	−7.7 (− 16.0; 0.6)
Educational level, years (≤12 vs. > 12)	− 3.2 (− 9.7; 3.2)	−0.5 (− 6.8; 5.9)	−2.8 (− 10.5; 4.8)	−3.0 (− 12.2; 6.3)	**− 10.0 (− 16.8; − 3.2)**	− 5.3 (− 12.6; 2.0)	− 4.1 (− 11.5; 3.3)	− 3.8 (− 11.4; 3.8)	6.1 (− 3.1; 15.2)	**− 11.5 (− 19.7; − 3.3)**
Occupation^a^										
Lower white collar vs. Upper white collar	− 4.7 (− 12.2; 2.8)	− 6.7 (− 13.8; 0.4)	−6.2 (− 15.0; 2.7)	−8.7 (− 19.3; 1.9)	**−12.1 (− 19.6; − 4.6)**	− 8.7 (− 19.4; 2.0)	−3.8 (− 15.0; 7.3)	−1.4 (− 12.7; 9.8)	−1.0 (− 14.8; 12.8)	−10.6 (− 22.7; 1.5)
Blue collar vs. Upper white collar	− 1.7 (− 11.1; 7.7)	2.0 (− 6.9; 10.9)	−1.8 (− 12.9; 9.3)	−6.8 (− 20.2; 6.6)	**−10.9 (− 20.4; − 1.5)**	−7.2 (− 14.7; 0.4)	−6.2 (− 14.1; 1.6)	−5.2 (− 13.1; 2.8)	3.1 (− 6.7; 12.8)	**−14.1 (− 22.6; − 5.6)**
Subjective Social Class (Low/Medium-low vs. Medium-High)	−4.0 (− 11.1; 3.2)	**− 9.2 (− 15.9; − 2.6)**	**− 9.1(− 17.3; − 0.8)**	− 9.1 (− 19.2; 1.0)	**− 15.7 (− 22.5; − 8.8)**	−1.0 (− 10.8; 8.8)	− 2.2 (− 12.2; 7.8)	−1.4 (− 11.6; 8.8)	9.0 (− 3.2; 21.2)	− 8.0 (− 19.5; 3.6)
Parenting stress										
Total stress scale	**− 0.1 (− 0.2; − 0.1)**	− 0.1 (− 0.2; 0)	**−0.2(− 0.3; − 0.1)**	**−0.2 (− 0.4; − 0.1)**	**−0.2 (− 0.3; − 0.1)**	−0.1 (− 0.2; 0)	−0.1 (− 0.2; 0)	**−0.2 (− 0.2; − 0.1)**	**−0.2 (− 0.3; − 0.1)**	−0.1 (− 0.2; 0)
Stressful Life Events scale^b^	**− 0.3 (− 0.6; − 0.1)**	−0.2 (− 0.4; 0.1)	−0.2 (− 0.5; 0.2)	−0.3 (− 0.7; 0.1)	**−0.4 (− 0.7; − 0.1)**	−0.1 (− 0.5; 0.2)	−0.3 (− 0.6; 0.1)	−0.2 (− 0.6; 0.2)	−0.3 (− 0.8; 0.2)	−0.4 (− 0.8; 0)
Anxiety	**−1.1 (− 1.6; − 0.6)**	**−1.3 (− 1.8; − 0.9)**	**−1.3 (− 1.9; − 0.7)**	**−1.4 (− 2.1; − 0.6)**	**−1.1 (− 1.7; − 0.5)**	**−1.1 (− 1.7; − 0.6)**	**−0.9 (− 1.5; − 0.3)**	**−1.2 (− 1.8; − 0.6)**	**−1.6 (− 2.4; − 0.9)**	**−1.1 (− 1.8; − 0.5)**
Depression	**−1.2 (− 1.8; − 0.7)**	**−1.6 (− 2.1; − 1.2)**	**−2.1 (− 2.6; − 1.6)**	**−2.0 (− 2.8; − 1.3)**	**−1.4 (− 2.0; − 0.8)**	**−1.3 (− 1.9; − 0.6)**	**−1.5 (− 2.2; − 0.9)**	**−2.2 (− 2.8; − 1.6)**	**− 1.9 (− 2.7; − 1.0)**	**−1.5 (− 2.3; − 0.7)**
Previous children (Yes vs. No)	−4.7 (− 12.0; 2.5)	0.3 (− 6.9; 7.4)	− 3.8 (− 12.5; 4.8)	0.3 (− 10.2; 10.8)	−4.6 (− 12.7; 3.5)	−1.5 (− 9.8; 6.8)	0 (− 8.5; 8.5)	−1.8 (− 10.5; 6.9)	−0.2 (− 10.5; 10.1)	−0.4 (− 10.3; 9.6)
Pregnancy complications^c^(Yes vs. No)	− 3.4 (− 9.9; 3.0)	1.2 (− 5.1; 7.5)	−3.8 (− 11.4; 3.8)	−0.9 (− 10.2; 8.4)	−2.9 (− 10.1; 4.3)	−0.7 (− 7.4; 6.1)	0.2 (− 7.0; 7.3)	4.8 (− 2.7; 12.3)	0.7 (− 8.3; 9.6)	2.0 (− 6.0; 10.0)
Multiple pregnancy (Yes vs. No)	5.7 (−2.3; 13.7)	− 4.8 (− 12.7; 3.0)	4.0 (− 5.6; 13.5)	−0.2 (− 11.8; 11.4)	3.3 (− 5.7; 12.3)	4.2 (− 4.2; 12.6)	3.5 (− 5.3; 12.4)	0.3 (− 9.1; 9.8)	2.8 (−8.3; 13.9)	0.1 (− 9.9; 10.0)
Extremely low birth weight delivery^d^ (Yes vs. No)	**−8.3 (− 15.0; − 1.5)**	0.7 (− 6.2; 7.6)	−0.5 (− 8.9; 7.9)	2.8 (− 7.3; 12.9)	3.7 (− 4.1; 11.5)	− 2.6 (− 10.2; 4.9)	−6.5 (− 14.3; 1.3)	−4.1 (− 12.4; 4.3)	−8.1 (− 17.8; 1.7)	−2.3 (− 11.2; 6.6)
Extremely preterm delivery^e^(Yes vs. No)	−3.1 (− 11.0; 4.7)	−2.9 (− 10.6; 4.8)	−2.1 (− 11.4; 7.2)	−1.9 (− 13.1; 9.4)	−3.1 (− 11.9; 5.6)	− 4.3 (− 12.7; 4.1)	0.7 (−8.2; 9.6)	−1.7 (− 11.2; 7.7)	−8.5 (− 19.4; 2.4)	−2.4 (− 12.4; 7.5)
Length of stay, months (≥2 vs. < 2)	**− 7.3 (− 13.7; − 0.8)**	− 2.9 (− 9.4; 3.7)	−5.2 (− 13.1: 2.7)	− 6.3 (− 15.8; 3.2)	−4.2 (− 11.6; 3.2)	−4.6 (− 11.4; 2.2)	**−9.9 (− 16.8; − 3.0)**	**−9.3 (− 16.6; − 1.9)**	**−12.0 (− 20.7; − 3.4)**	− 6.0 (− 14.1; 2.1)
Infants’ health problems^f^(Yes vs. No)	**−8.3 (− 15.5; − 1.1)**	−5.3 (− 12.5; 1.9)	−6.2 (− 14.9; 2.5)	**−** 7.7 (− 18.2; 2.8)	−5.5 (− 13.7; 2.7)	−1.8 (− 10.6; 6.9)	−6.2 (− 15.3; 2.9)	− 4.1 (− 13.8; 5.7)	**−13.3 (− 24.3; − 2.3)**	**−11.8 (− 21.6; − 2.0)**
Urbanity Level (Predominantly Rural/Moderately Urban vs. Predominantly Urban)	−8.5 (− 17.8; 0.8)	−8.8 (− 17.8; 0.3)	− 9.8 (− 20.8; 1.2)	−10.5 (− 23.9; 2.9)	−8.4 (− 18.8; 2.1)	− 5.6 (− 15.6; 4.4)	−0.9 (− 11.6; 9.8)	− 6.5 (− 17.7; 4.7)	−0.5 (− 13.9; 12.9)	−10.0 (− 21.7; 1.6)
Neighbourhood socioeconomic deprivation^g^										
T2 vs. T1	−1.1 (− 8.6; 6.4)	−3.9 (− 11.1; 3.3)	− 7.9 (− 16.6; 0.7)	−6.2 (− 16.8; 4.4)	− 5.9 (− 14.1; 2.3)	4.2 (− 3.7; 12.0)	− 1.8 (− 10.2; 6.6)	3.8 (− 5.0; 12.6)	9.8 (− 0.3; 19.8)	− 0.7 (− 10.1; 8.7)
T3 vs. T1	−2.2 (− 10.6; 6.1)	2.7 (− 5.3; 10.7)	− 4.8 (− 14.4; 4.8)	− 3.7 (− 15.5; 8.1)	−2.7 (− 11.9; 6.5)	4.9 (− 3.4; 13.2)	1.2 (− 7.6; 10.0)	3.8 (− 5.6; 13.1)	9.9 (− 0.8; 20.5)	0.5 (− 9.4; 10.5)

^a^Students, housewives and armed forces occupations were excluded; ^b^Stressful Life Events scale is composed by 24 different life events likely to cause stress (e.g.: unemployment, divorce, death of a relative); ^c^Infectious, placental, haemorrhagic and cardiovascular complications; ^d^ < 1000 g; ^e^ < 28 gestational weeks; ^f^Inguinal and umbilical hernias, metabolic disease, ovarian cysts, bronchial dysplasia, autoimmune disease, cardiac disease, congenital malformation; ^g^From tertile 1 (T1) (least deprived) to tertile 3 (T3) (most deprived)

Notes: 95% CI, 95% confidence interval; Bold type indicates statistically significant associations (*p* value < .05)

### Overall QoL and accommodation mechanisms

A quantitative analysis revealed that the perception of overall QoL was not significantly different by gender (Mean (SD): 74.6 (12.5) for mothers; 72.8 (12.4) for fathers). Higher levels of anxiety and depressive symptoms were negatively associated with the parental perception of overall QoL. Among mothers, having higher levels of total stress, higher stress life scores, an extremely low birth weight delivery and an infant with health problems or hospitalized in NICU for 2 months or more, was associated with worst overall QoL.

Four main mechanisms to accommodate the delivery of a very preterm infant on their lives were mentioned by the interviewed couples. Firstly, being optimistic by choosing to *“be very practical”* and *“to think positive” (I26)*, despite being scared:

*“Despite these little scares [cold, urinary tract infection and conjunctivitis], everything is going positively, it is going well.” (I18)*

Secondly, reordering goals by giving priority to the infant and learning to devalue stressful *“little things”* while attributing more value *“to the really important things”* such as seeing the infant breathing autonomously:

*“The little things that stress us on daily life (…) nowadays we devalued it, we attribute more value to the really important things (...) [like] seeing him [son] breathing for himself [without medical support] every day.” (I2)*

Thirdly, using comparisons between their infants and those with severe health problems to highlight how they are *“lucky” (I2)* and should *“thank God” (I24)*. Lastly, reframing expectations about the current and future development of their infant helped parents to deal with the experience of parenting a very preterm infant:

*“He [son] had some little problems (…) but it’s nothing of concern in terms of development. (…) We can’t expect that he, with 4 months, matches with a 4 months term baby.” (I11)*

*“In the future, it [the concern] will be knowing if she [daughter] will develop the speaking skill (…) the growth we already know that it will be slow.” (I13)*

### Physical QoL

Based on a quantitative analysis, physical QoL was slightly higher among fathers (Mean (SD): 78.3 (13.1) vs. 75.9 (12.2) for mothers). This perception was negatively associated with higher levels of anxiety and depressive symptoms among mothers and fathers. Physical QoL was lower among mothers from a lower subjective social class and among fathers of infants hospitalized in NICU for 2 months or more.

Interviewees only mentioned negative factors influencing their physical QoL. The main issues presented by the parents included sleep deprivation, nightmares and poor sleep quality, as well as unpredictability and lack of time to perform daily activities or organizing the house. Some interviewees considered the infant’s dependence on medical substances and medical aids as *“a daily challenge” (I19)*, and reported self-dependence of medication to manage headaches connected to the burden of parenting very preterm infants (I25). Few couples emphasized the deterioration of working capacity by feeling *“lost [and] disorientated”* to supervise employees *(I17)*, as well as the discomfort experienced when pumping breast milk, seen as a *“little sacrifice”* for the child *(I11)*, and the tiredness provoked by the intensive full-time caring of a very preterm infant:

*“It’s like a 24 out of 24 hours job and then the tiredness is different. (…) Because she is preterm, [the routine is] even more intense.” (I24)*

### Psychological QoL

The highest quantitative score among the parental QoL domains was observed in the psychological domain among both mothers (Mean (SD): 77.2 (14.8)) and fathers (Mean (SD): 80.4 (13.9)). Lower levels of psychological QoL were associated with higher levels of parenting stress, anxiety and depression, for both mothers and fathers. This domain was also negatively associated with mother’s lower subjective social class, and with having an infant hospitalized in NICU for 2 months or more among fathers.

Interviewees mentioned the surveillance as a major constraining factor to psychological QoL. Parents were aware of the burden caused by surveillance but revealed difficulties in overcoming their *“instinctive”* need to control all social interactions established with the baby and the environment, as well as their distrust on relatives and friends to take care of the infant:

*“[When other people hold my son] I usually stay like “a security dog” (…) it’s like an instinct.” (I16)*

*“The environment is always controlled. (…) The house has to be clean every day. (…) We have thermometers all over the house.” (I2)*

*“I can’t leave my daughter (…) with anybody. (…) I don’t know why.” (I6)*

Participants justified such difficulties by expressing negative feelings that involve fears and uncertainties around the return to the hospital, the infant’s death or suffering or the infant’s future development. A few interviewees also invoked thinking difficulties, a *“completely loss of personal autonomy”* related to the need to live according to their infants *(I11)* and mixed emotions:

*“It’s a whirlwind of emotions, and it’s a challenge dealing with all that things.” (I24)*

*“[Having a very preterm infant] means happiness, means torment, anxiety and joy.” (I25)*

Some respondents neutralized the negative influence of a very preterm delivery on psychological QoL by focusing on positive feelings, such as *“joy”* and *“happiness”*, and assuring self-esteem based on self-confidence as *“strong”* women and *“very careful and responsible”* mothers. Additional strategies were related with enacting spirituality/religion and personal beliefs (e.g. considering that *“things happen because they have to happen, and we have to face them” (I1)*), as well as acquiring parental autonomy by learning how to administrate medical treatments at home:

*Father: “We are more self-sufficient if we do the things [administrate injections] at home, so I have learnt to give the injection. We don’t need to go out with him [son] to do this medical treatment.”*

*Mother: “Neither we are dependent of other people.” (I24)*

### Social relationships QoL

Mothers and fathers presented similar values of social QoL (Mean (SD): 75.8 (17.9); 74.3 (16.4), respectively). This domain was negatively associated with higher levels of parenting stress, anxiety and depressive symptoms among women and men. Fathers of infants hospitalized for 2 months or more and with health problems presented lower levels of social QoL.

In interviews, parents mentioned the benefits of pragmatic or emotional support provided by family, friends, healthcare providers or other parents of very preterm infants:

*“We have my parents-in-law, and sometimes my parents, helping us to take care of him [son], for allowing us to do other things [washing the car, rest].” (I26)*

*“Now they [friends] are [acting] with normality, they are more positive (…) They try to relax us and transmit us security.” (I21)*

*“If we [parents] don’t know what to do we can call the NICU professionals of where he [son] was [hospitalized] (…) anytime.” (I8)*

*“Sometimes we [parents] call them [other NICU parents] and ask them how they dealt with baby’s cramps. We talk to each other a lot of times.” (I25)*

Different perspectives toward personal relationships were reported: some couples stated that the very preterm childbirth strengthen their marital relationship, while others complained about the lack of time *“for each other”*. Likewise, parents distinguished between supportive personal networks and those who criticize them:

*“We feel a great understanding about our concerns with hygiene, I think we always felt they [family and friends] understand us and that they do everything to facilitate [our life].” (I12)*

*“We know that (…) a lot of people and family members criticize us because we are excessively careful [with the infant].” (I3)*

### Environment QoL

The lowest quantitative scores on parental QoL were observed in the environment domain (Mean (SD): 72.9 (13.9) among mothers; 71.3 (14.6) among fathers). They were negatively associated with lower levels of education, having blue-collar occupations and higher levels of anxiety and depressive symptoms for both parents. Among mothers, lower scores of environment QoL were also associated with having lower white-collar occupations, a low/medium-low subjective social class, higher levels of parenting stress and higher stress life scores. Fathers of infants with health problems scored worst on environment QoL.

Interviewees focused on the influence of the accessibility and quality of health and social care. Parents recognized government financial support for infant’s healthcare, namely for hospitalization, medication and vaccination, and their satisfaction with medical services as enabling factors, but pointed the negative influence of non-supportive parental leave policies and family allowance, as well as lack of coverage of *“special”* milk and all vaccines that preterm babies need and the absence of a *“fast track”* for very preterm infants in the emergency room.

*“Due to the infant prematurity, the parental leave should be extended, for both mother and father. (…) I would start working next month and she [daughter] needs special care at least for one year.” (I20)*

Some participants also mentioned the negative influence of the hygienization of bodies and spaces. The concern with the sterilisation of hands and objects and the avoidance of touch and closeness in the relationships with the infant adversely affected their QoL:

*Father: “The care with sterilization of hands (…) [and] for not kissing him [son] - perhaps if he was a normal baby there are things that we didn’t going through.”*

*Mother: “If something drops to the floor, it goes immediately to the laundry.” (I2)*

*“At the entrance room, they [visits] have to put the mask on and to wash and sterilise the hands and they’re only allowed to see the baby, nobody can touch her [daughter].” (I20)*

Other issues presented by the interviewees included constraints on their participation in recreation and leisure activities. They often referred to isolation and the absence of a *“social life”* as threatening their QoL. A few participants overcome these by taking advantage of opportunities to share *“enjoyable”* moments together, such as *“watching a movie or talking to each other” (I12).* A few couples also reported different perspectives regarding the home environment and financial resources by combining their negative and positive influence on QoL in a hybrid way. The need to rearrange small home spaces due to their infant’s medical needs, like *“a medical oxygen cylinder” (I19)*, to become aware of their family’s inability to fulfil infant’s needs due to financial constraints and lack of support in transportation *“for taking the infant to the clinical appointments” (I9)* contributed to deteriorate QoL, while the access to conditions for creating a *“calm environment”* in the household and to financial resources positively affected QoL.

Some participants highlighted how the opportunities for acquiring new information and skills improved their sense of competence and control at home. These opportunities occurred either during infant’s hospitalisation in NICU through the *“intensive course”* provided by health professionals or outside NICU by being offered the opportunity to clarify doubts about the baby by the paediatrician:

*“We learned a lot [in NICU]. (…) It was there the father changed the first diaper, gave the first bath… He came home very prepared. (…) We used to say that it was an intensive course.” (I18)*

*“For us the most important thing is (...) having a person [health professional] to contact (…) anytime to clarify our doubts.” (I5)*

**Fig. 1:**
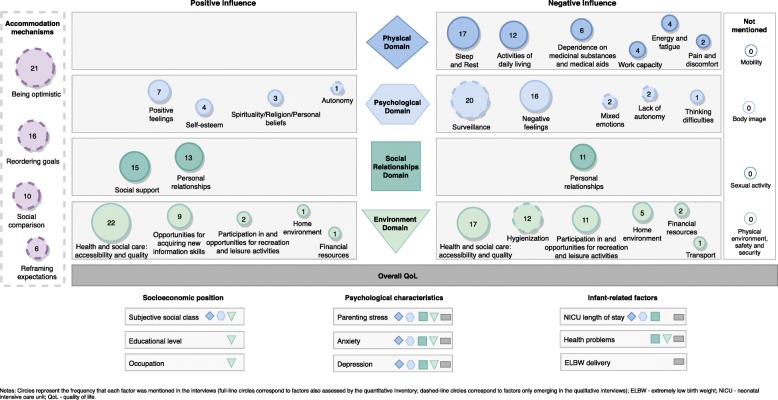
Map of the factors influencing the QoL of parents of very preterm infants

## Discussion

Quantitative data suggest that mothers and fathers of very preterm infants present similar values of QoL, increasing slightly from the environment to the psychological domain. Parenting stress, anxiety or depressive symptoms negatively influence both maternal and paternal QoL, while the impact of socioeconomic position and infant-related factors (NICU length of stay, health problems and extremely low birth weight delivery) varies according to gender and QoL domains. Qualitative findings highlight constraining factors related with surveillance, non-supportive healthcare policies and the need for hygienization, and protective factors as social support, accessibility and quality of healthcare, and opportunities for developing parental skills.

Participants’ quantitative assessment of QoL is comparable to the scores observed in the Portuguese general population [32, 42, 43], reinforcing previous findings showing that there are no differences in QoL between parents of very low birth weight infants and the general population [44]. The negative association between depressive [11] or psychiatric symptoms [10] and QoL among mothers and caregivers of preterm infants has also been reported previously, as well as the influence of socioeconomic position [45]. Moreover, the stress-buffering effects of social support [46] and the positive impact of family-friendly and gender-equality policies [9, 47] on QoL are widely recognized.

This study adds to the literature the idea that similar quantitative scores of QoL might hide social inequalities and translate different meanings behind QoL. Figure 1 represents a comparison matrix in which a side-by-side joint display is used to converge the quantitative and qualitative data. First, each item assessed by the quantitative instrument does not acquire the same relative weight in parents’ narratives. Second, there are facets assessed by the survey not mentioned by interviewees (mobility, body image, sexual activity, physical environment, safety and security). Third, parents mention several issues during the interviews that are not addressed by the questionnaire (such as the constant surveillance, hygienization of bodies and spaces, experience of mixed emotions, and lack of autonomy as negatively influencing their QoL). Bringing together the differing but complementing strengths of quantitative methods (e.g., trends and generalization) with those of qualitative methods (e.g. in-depth description and details) in a one-phase design might thus contribute to develop a specific quantitative tool to sensitively assess QoL of parents of very preterm infants, while helps to better understand their underlying factors.

When experiencing a very preterm childbirth, parents adjusted their expectations and changed their internal standards to accommodate such a catalyst event in their lives [21], as reported in studies with chronic illnesses patients [48]. The accommodation mechanisms observed in this study (being optimistic, reordering goals, social comparison and reframing expectations) are anchored in child-centredness, reflecting the incorporation of intensive parenting social norms and leading to the prioritization of child’s health and well-being over parents’ QoL [49], and in a pragmatic framework that construct hierarchies of hope [44] and expectations about infant’s health status and development.

Couple interviews may have limited emergence of some facets assessed by the survey, in particular those related with body image and sexual activity. Interviewed parents may have felt uncomfortable acknowledging these issues in couple. In addition, the possibility of assuming as taken for granted facets as physical environment, safety and security cannot be excluded, as demonstrated by the quantitative rates. Further studies should explore the meanings attributed to each of these facets, discussing the implications for the assessment of the QoL.

These achievements reinforce the idea that the use of generic instruments may not be sensitive enough to accurately capture the specificities and idiosyncrasies of parents of very preterm infants [25, 50], overestimating their QoL. However, to acknowledge an individual holistic assessment that considers spirituality, religion and personal beliefs in QoL measurements is a step forward to improve the sensitivity of quantitative instruments, especially in health context [51–53]. Still, there is a need for further research on the development of a new quantitative tool specifically designed for being used to assess QoL among parents of very preterm infants.

The use of a convergent mixed methods design is a strength of this study, in which the inclusion of researchers who have quantitative and qualitative expertise addressed the effort to offer equal weight to two type of data. The sample size and the response rate could limit the power to detect small but potentially important differences, but they are quite similar to those observed in other studies with comparable populations and objectives [14, 54]. Moreover, there are no significant differences between participants who returned the questionnaire and those who did not regarding all the assessed variables except for marital status. Participants are more likely to be married or living with a partner (92.1% vs. 82.9% among non-participants, *p* = .044), which could cause some bias, since married people are more likely to score higher in the QoL questionnaires than people with other marital status [14, 55].

## Conclusions

This study raises awareness for the need to capture the QoL of parents of very preterm infants using a mixed-methods approach for developing intersectoral family-centred public policies, integrated healthcare services on prematurity and focused-interventions to decrease the disempowering effects of surveillance and hygienization.

## Abbreviations

NICU: Neonatal Intensive Care Unit
QoL: Quality of Life
WHOQOL-BREF: World Health Organization Quality of Life – BREF Inventory
